# Asialoglycoprotein receptor 1: a multifaceted receptor in the liver and cardiovascular system

**DOI:** 10.3389/fmed.2025.1653452

**Published:** 2025-08-07

**Authors:** Xuan Xiao, You Nie, Yiping Leng

**Affiliations:** ^1^Department of Laboratory Medicine, Hengyang Medical School, The Affiliated Changsha Central Hospital, University of South China, Changsha, China; ^2^Hengyang Medical School, The Affiliated Changsha Central Hospital, Changsha Tuberculosis Research Institute, University of South China, Changsha, China

**Keywords:** ASGR1, glycoprotein recognition, viral infection, lipid metabolism, hepatocellular carcinoma, targeted drug delivery

## Abstract

Sialic acid is a common terminal monosaccharide residue on glycan chains, and desialylation of glycoproteins is considered an important biological signal. In the liver and other cell types, asialoglycoprotein receptor 1 (ASGR1) specifically recognizes and binds to exposed galactose or N-acetylgalactosamine (Gal/GalNAc) residues on desialylated glycoproteins, and activates downstream signaling pathways through receptor-mediated endocytosis (RME), thereby playing important roles in various physiological and pathological processes such as immune regulation, viral infection, hepatocellular carcinoma progression, and lipid metabolism. In addition, ASGR1 is regarded as a key target for liver-specific drug delivery. This review systematically describes the molecular structure and physiological functions of ASGR1, its roles in pathological processes, and its potential functions in extrahepatic tissues. It provides essential background information for a comprehensive understanding of ASGR1 and offers novel insights into future research directions.

## 1 Introduction

The central role of the liver in body metabolism frequently exposes hepatocytes to drugs, microbes, and toxins that may cause various liver diseases (1). Asialoglycoprotein Receptor 1 (ASGR1) was discovered in the 1960s and was initially regarded as a clearance-type hepatic receptor. It has since been extensively studied due to its ability to recognize glycoproteins with terminal galactose (Gal) or N-acetylgalactosamine (GalNAc) residues (2, 3). However, recent studies have revealed that ASGR1 functions extend far beyond traditional understanding, emerging as a critical molecule linking immune regulation (4), viral infection (5), tumor progression (6), lipid metabolism (7), and targeted drug delivery (8). Currently, the molecular mechanisms and precise modes of action for these functions remain incompletely understood. Particularly unclear are the differential roles of ASGR1 across various tissues and pathological states, and its potential in drug delivery applications. Therefore, this review systematically summarizes the latest research progress on ASGR1, providing a comprehensive overview of its molecular structure and biological functions, physiological roles, pathological mechanisms, and future research directions.

## 2 The structure and biological functions of ASGR1

### 2.1 Protein structure of ASGR1

**Figure 1:**
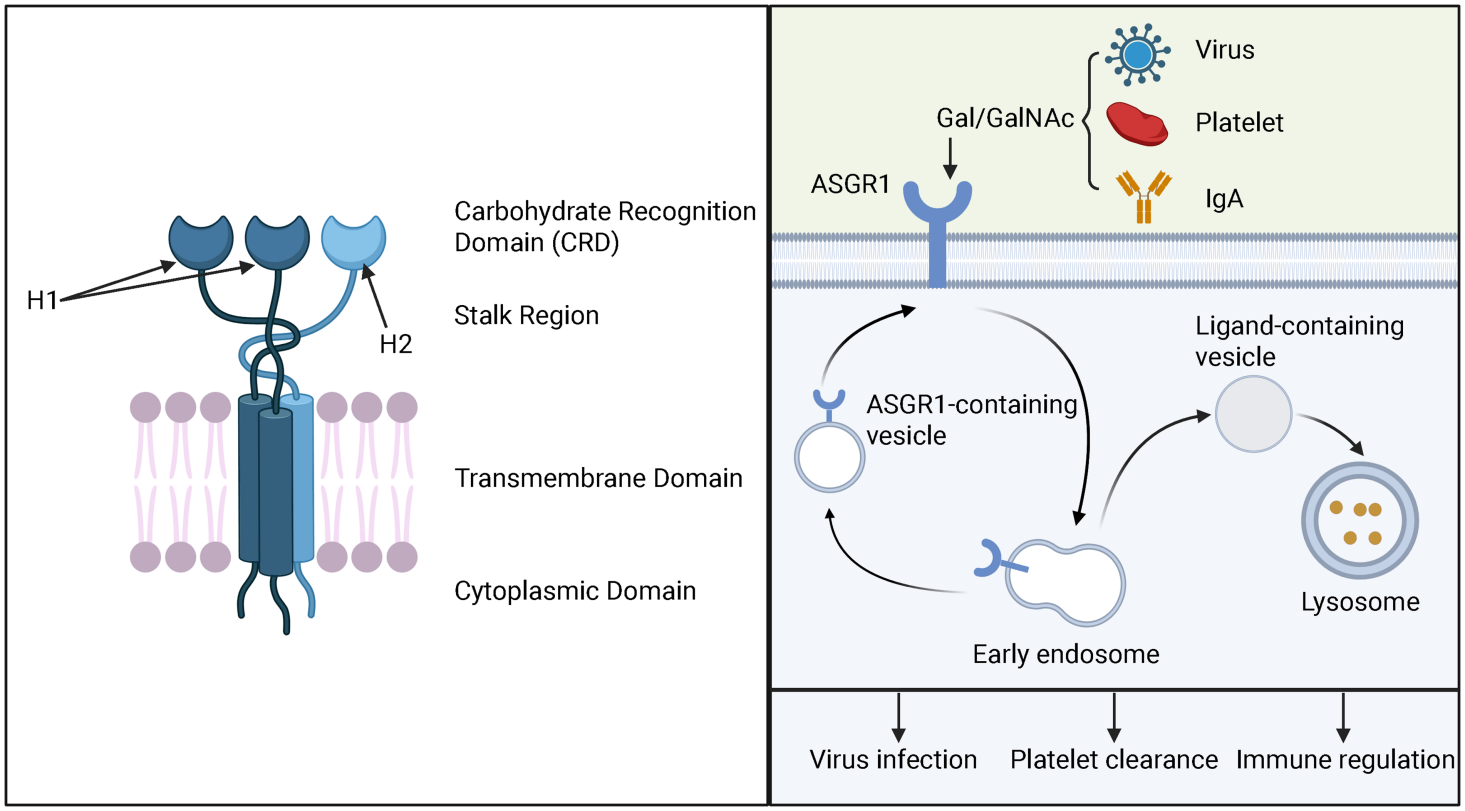
ASGPR receptor structure (Left) and ASGR1-mediated endocytosis process and its biological functions (Right). The left panel illustrates the schematic structure of ASGPR, highlighting its heterooligomeric composition consisting of two H1 subunits and one H2 subunit. Each subunit consists of four domains: a short N-terminal cytoplasmic domain, a transmembrane region, an extracellular stalk region, and a calcium-dependent CRD. The right panel depicts the ASGR1 endocytosis and recycling process, along with its biological functions in viral infection, platelet clearance, and immune regulation.

The Asialoglycoprotein Receptor (ASGPR) was the first mammalian lectin discovered, initially identified by Ashwell and Morell and colleagues through their research on mammalian plasma glycoprotein metabolism (2, 3). The human ASGPR gene is located on chromosome 17 (9) and consists of a hetero-oligomeric complex of two homologous but structurally distinct subunits, named ASGR1 (H1 subunit, approximately 46 kDa) and ASGR2 (H2 subunit, approximately 50 kDa). Each subunit is a type II single-pass transmembrane protein, composed of a short N-terminal cytoplasmic domain (~40 amino acids), a transmembrane region (~20 amino acids), an extracellular stalk region (~80 amino acids), and a calcium-dependent Carbohydrate Recognition Domain (CRD, ~140 amino acids) (10, 11). Among the two subunits, ASGR1 (H1 subunit) is widely regarded as the primary functional subunit, mainly responsible for ligand recognition and mediating the endocytosis process (12–14). H2 itself lacks the ability to bind glycan ligands and is unstable when expressed alone, leading to rapid degradation (15). However, the H2 subunit is not redundant; rather, it plays a crucial role in high-affinity ligand binding. It is thought to act as a structural scaffold or bridge that helps organize multiple CRDs into an optimally spaced recognition platform for ligand binding (13) (Figure 1). ASGR1 and ASGR2 can form various receptor complexes, including ASGR1–ASGR2 heterooligomers, ASGR1 homooligomers, and ASGR2 homooligomers. These different oligomeric forms on the plasma membrane provide the structural basis for substrate recognition and endocytosis (16). Each form exhibits distinct ligand-binding specificities, suggesting that modulation of ASGPR oligomerization may be a potential strategy to diversify its ligand recognition capabilities and enable targeted therapeutic applications for related diseases (13, 16).

ASGR1 is primarily expressed on the surface of hepatic parenchymal cells, with each cell containing about (1−5) ×10^5^ binding sites (17–19). Its classical physiological function is to recognize terminal galactose (Gal) and N-acetylgalactosamine (GalNAc) residues in a calcium-dependent manner through its extracellular CRD (2, 3). The high-affinity binding between ASGR1 and glycoproteins depends on calcium ions (Ca^2+^), which enhance the thermal stability of ASGR1 and prevent denaturation (3, 20, 21). Structural studies have shown that calcium ions coordinate with conserved amino acid residues (such as glutamate and asparagine) within the CRD, stabilizing carbohydrate binding. These interactions enable ASGR1 to form hydrogen bonds and coordination bonds with the 3- and 4-hydroxyl groups of Gal/GalNAc, thereby conferring high specificity (22). However, the affinity between a single CRD and a monosaccharide is relatively weak. Therefore, multivalent ligands, by simultaneously engaging multiple CRDs, can significantly enhance binding strength and increase overall affinity (23, 24). An important feature of protein–glycan interactions is multivalency (23). In addition to enhancing binding affinity (25), multivalency can also greatly improve binding selectivity. So-called super-selective binding occurs when the density of the target ligand on the cell surface exceeds a certain threshold (26, 27), enabling discrimination between target cell types that display different numbers of specific glycans or lectins, and laying the foundation for the exceptional liver-targeting specificity of GalNAc-conjugated therapeutics (28).

Multivalency has also been shown to influence the mechanism of ligand internalization. Nanoparticles with low ligand density tend to enter cells via caveolae-mediated endocytosis, which directs them toward perinuclear compartments such as the Golgi apparatus and endoplasmic reticulum, thereby avoiding lysosomal degradation. In contrast, nanoparticles with high ligand density predominantly enter cells via clathrin-mediated endocytosis and are delivered to lysosomes for degradation (29, 30).

### 2.2 Receptor-mediated endocytosis of ASGR1

For ASGR1, its endocytic mechanism specifically belongs to receptor-mediated endocytosis (RME) and is classified as clathrin-mediated endocytosis (31, 32). Notably, neither H1 nor H2 subunit alone is sufficient for ligand binding or internalization; co-expression is essential (15). ASGR1-mediated endocytosis is extensively studied, with receptor internalization half-life of approximately 5–6 min without ligands, accelerated to 2.5–3 min in the presence of ligands, followed by receptor recycling to the cell surface within 5–7 min (33). This efficient cycle involves clathrin-coated pits-mediated endocytosis, ligand dissociation in early endosomes, receptor recycling to the plasma membrane, and ligand degradation in lysosomes, a process known as RME (31, 32, 34). ASGR1 surface levels are regulated by endocytosis and recycling efficiency (35, 36) (Figure 1).

### 2.3 Physiological functions of ASGR1

The ligands recognized by ASGR1 possess specific glycan features. ASGR1 belongs to the C-type lectin family, which typically recognizes sugar residues such as mannose or galactose; however, ASGR1 specifically binds to terminal galactose (Gal) and N-acetylgalactosamine (GalNAc) residues (37, 38). Glycans are crucial components for endogenous glycoprotein homeostasis and circulating clearance. Modulating glycan patterns can alter glycoprotein concentration and half-life, thereby changing their clearance rate from circulation (39). A key factor in plasma glycoprotein clearance is desialylation, where sialic acid residues are removed by circulating sialidases (NEU1, NEU3, NEU4) (40). This process exposes terminal Gal or GalNAc residues, making them recognizable by ASGR1, thus initiating glycoprotein clearance (41). Interestingly, α2,3-sialylation abolishes ASGR1 affinity, whereas α2,6-sialylation does not (42). O-acetylation of sialic acids protects them from desialylation by reducing sialidase activity (43, 44).

Although the role of ASGR1 in clearing desialylated glycoproteins is confirmed, its overall impact on circulating glycoprotein metabolism remains debated (45). A mouse glycomics study revealed no significant accumulation of terminally desialylated or galactose-exposed glycoproteins in ASGR1 deficiency, suggesting compensatory regulatory mechanisms (46). Thus, the critical role of ASGR1 in glycoprotein homeostasis remains inconclusive.

Beyond these classical functions, recent studies indicate the involvement of ASGR1 in various biological processes. For example, ASGR1 may play roles in immune regulation (47). Moreover, recent studies have revealed that ASGR1 also participates in lipid metabolism regulation (48, 49).

Several studies have identified ASGR1 homologs in various non-hepatic cell types, such as DC-ASGPR and Rat Hepatic Lectin-1 (RHL-1) (50, 51). These proteins exhibit a certain degree of structural or functional similarity to hepatic ASGR1 (52, 53). It should be noted that the hepatic-type ASGPR expressed in Caco-2 cells (54), the ASGPR-like receptor in human renal proximal tubular epithelial cells (RPTEC) (55), and RTG-r in rat testis (which is antigenically related to RHL-2/3) (56) currently lack comprehensive amino acid sequence comparisons. Therefore, whether these proteins are truly homologous to hepatic ASGR1 remains to be further investigated.

## 3 Pathophysiological roles of ASGR1

### 3.1 Multifaceted roles of ASGR1 in immune regulation

C-type lectin receptors (CLRs) play a crucial role in the activation of innate immunity (38). Acting as pattern recognition receptors (PRRs) and endocytic receptors, CLRs recognize pathogen-associated glycan structures, facilitating pathogen-receptor complex internalization and activating innate immune responses (57). ASGR1, as a C-type lectin receptor, is expressed in DCs (38). Studies have shown that the expression and function of glycan-recognizing receptors differ between immature and mature DCs, DC-ASGR1 is highly expressed only in immature DCs and is rapidly downregulated upon maturation, indicating a close association between DC-ASGR1 and the maturation status of DCs. However, whether DC-ASGR1 directly regulates dendritic cell maturation requires further investigation (52).

Studies have shown that ASGR1 mediates the differentiation of monocytes into macrophages (58). In the LPS (Lipopolysaccharide)-induced septic mouse model, ASGR1 promotes the phosphorylation of NF-κB (Nuclear Factor kappa-light-chain-enhancer of activated B cells) and IκBα (Inhibitor of kappa B alpha). After the phosphorylation of P65 enters the nucleus, it increases the expression of ATF5 (Activating Transcription Factor 5), thereby promoting the differentiation of monocytes into macrophages, elevating the levels of pro-inflammatory cytokines (such as TNF-α, IL-6, IL-1β), and exacerbating liver inflammation (58). Current studies suggest that ASGR1 may act as an upstream regulatory factor promoting the differentiation of pro-inflammatory macrophages, thereby contributing to the onset and progression of inflammatory liver injury. Moreover, in nonalcoholic steatohepatitis (NASH) and other chronic liver diseases, sustained macrophage-driven inflammation is considered a key driver of fibrosis progression (59). The ASGR1-mediated monocyte-to-macrophage differentiation mechanism may also play a pathological role in such conditions. Therefore, targeting ASGR1 or its associated signaling axis holds promise as a novel strategy for controlling hepatic inflammatory responses and blocking macrophage polarization, and warrants further investigation in broader models of inflammatory liver disease.

Apart from its role in immune cells, ASGR1 is closely related to endoplasmic reticulum (ER) stress during liver injury (60). ASGR1 binds to the circulating liver disease biomarker Golgi Protein 73 (GP73) and mediates its endocytosis for subsequent lysosomal degradation. In the absence of ASGR1, elevated levels of GP73 interact with the ER stress marker immunoglobulin heavy chain binding protein (BIP), leading to the activation of ER stress pathways and ultimately exacerbating liver injury (60). Under non-stress conditions, the endoplasmic reticulum (ER) stress marker BIP forms complexes with the three major sensors—inositol-requiring enzyme 1 (IRE1), protein kinase RNA-like endoplasmic reticulum kinase (PERK), and activating transcription factor 6 (ATF6)—thereby maintaining these signaling factors in an inactive state (61, 62). Upon cellular stress, BIP dissociates from these sensors, leading to the activation of the unfolded protein response (UPR) (62, 63). This suggests that GP73 is no longer merely a traditional liver disease biomarker (64), but may also act as a pathogenic contributor and potential therapeutic target. As a key regulatory pathway of cellular stress and injury, the UPR plays a critical role in the progression of various liver diseases (65). Targeting the UPR pathway may offer a novel approach for precision therapy in chronic liver diseases.

**Figure 2:**
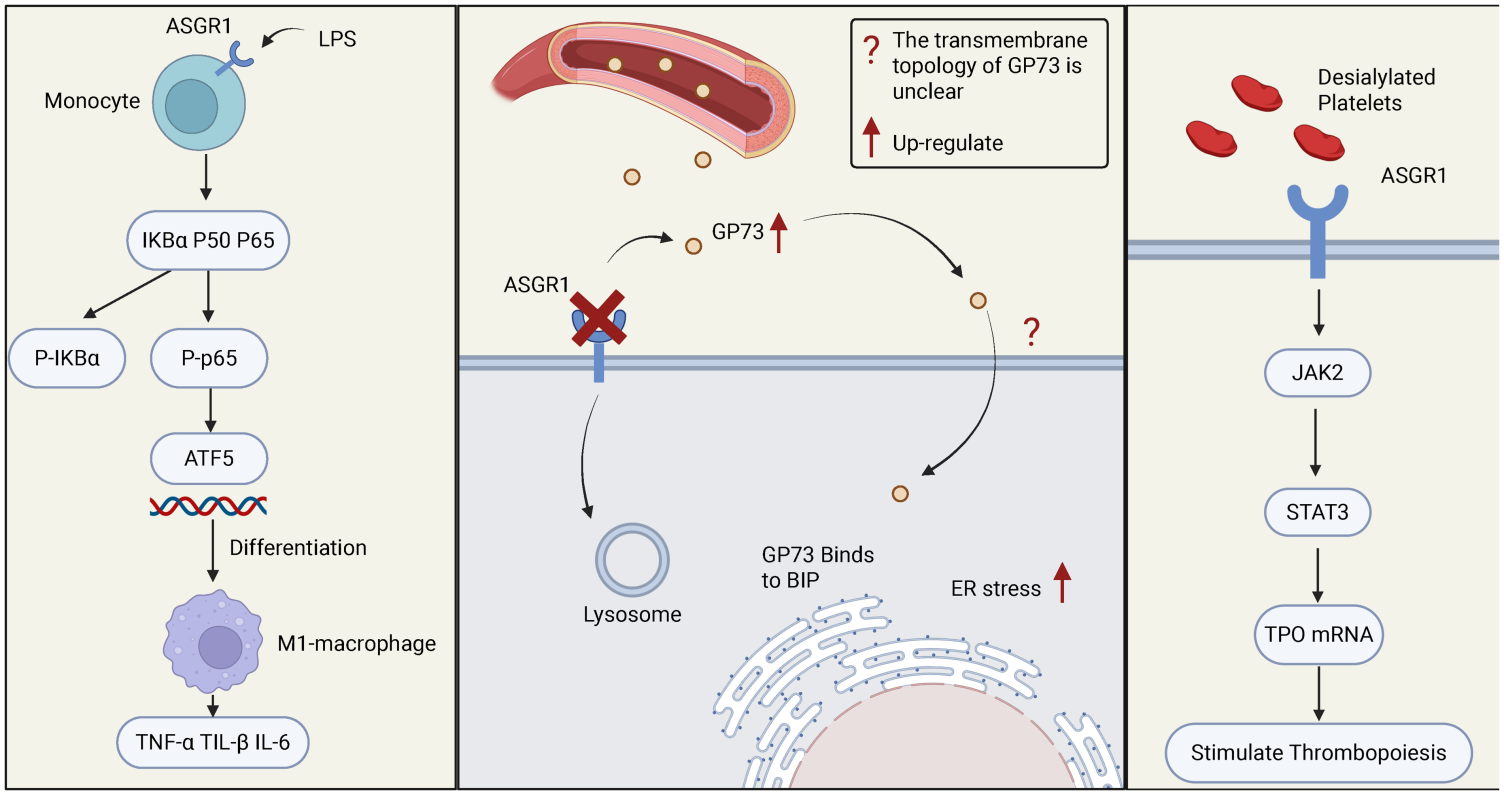
The role of ASGR1 in relevant immune responses. The left panel shows how ASGR1 regulates the differentiation of monocytes into macrophages through the NF-KB/ATF5 pathway, leading to elevated levels of pro-inflammatory cytokines (such as TNF-α, IL-6, IL-1β) and exacerbating liver inflammation. The middle panel demonstrates the role of ASGR1 in ER stress, where ASGR1 deficiency increases GP73-mediated liver ER stress, promoting liver injury. The right panel shows the regulatory role of ASGR1 in thrombopoiesis, where ASGR1 modulates liver thrombopoietin production through the JAK2-STAT3 signaling pathway.

In addition to its roles in immune cell function and stress regulation, ASGR1 also mediates the clearance and rebalancing of blood components, highlighting its unique contribution to the regulation of blood immune homeostasis. ASGR1 recognizes and clears desialylated platelets, a process that not only participates in platelet clearance but also stimulates hepatic thrombopoietin (TPO) synthesis via the janus kinase/signal transducer and activator of transcription 3 (JAK2/STAT3) pathway, maintaining platelet homeostasis (66). Clinically, thrombocytopenia is a common complication in patients with chronic liver disease (67). ASGR1 agonists or multivalent GalNAc ligands may be used to enhance TPO expression, thereby improving liver disease–associated thrombocytopenia. Meanwhile, the expression level or functional status of ASGR1 may also serve as a potential biomarker for predicting platelet production capacity. Additionally, studies in pig models indicate ASGR1 expression beyond the liver, such as peripheral vascular endothelial cells (e.g., aorta, femoral artery), recognizing human platelet surface-exposed Gal/GalNAc residues, leading to platelet phagocytosis. This mechanism may contribute to severe thrombocytopenia in xenotransplantation (human-to-pig transplantation) (68) (Figure 2).

In addition, ASGR1 is involved in the clearance of adaptive immune products, highlighting its unique role in maintaining humoral immune homeostasis. Studies have shown that ASGR1 participates in the clearance of circulating IgA; however, it remains controversial whether this process is primarily mediated through the recognition of N-glycans or O-glycans on the IgA1 subtype (69, 70). ASGR1 is also involved in the clearance of circulating low-density lipoprotein (LDL) (71, 72), chylomicron remnants (73), and cellular fibronectin (74).

### 3.2 The role of ASGR1 in viral infections

Early studies indicated ASGR1 specifically binds hepatitis B virus (HBV) particles, mediating hepatic endocytosis and potentially facilitating hepatocyte infection by HBV (75). Numerous studies confirm that ASGR1 enhances HBV infectivity by binding to the viral preS1 region, facilitating viral uptake into hepatocytes. This interaction represents a key mechanism in liver-specific HBV infection, underscoring the role of ASGR1 in viral entry (76, 77). Similarly, ASGR1 facilitates hepatitis E virus (HEV) infection through interaction with viral open reading frame 2 (ORF2) protein, enhancing hepatocyte entry (78).

**Figure 3:**
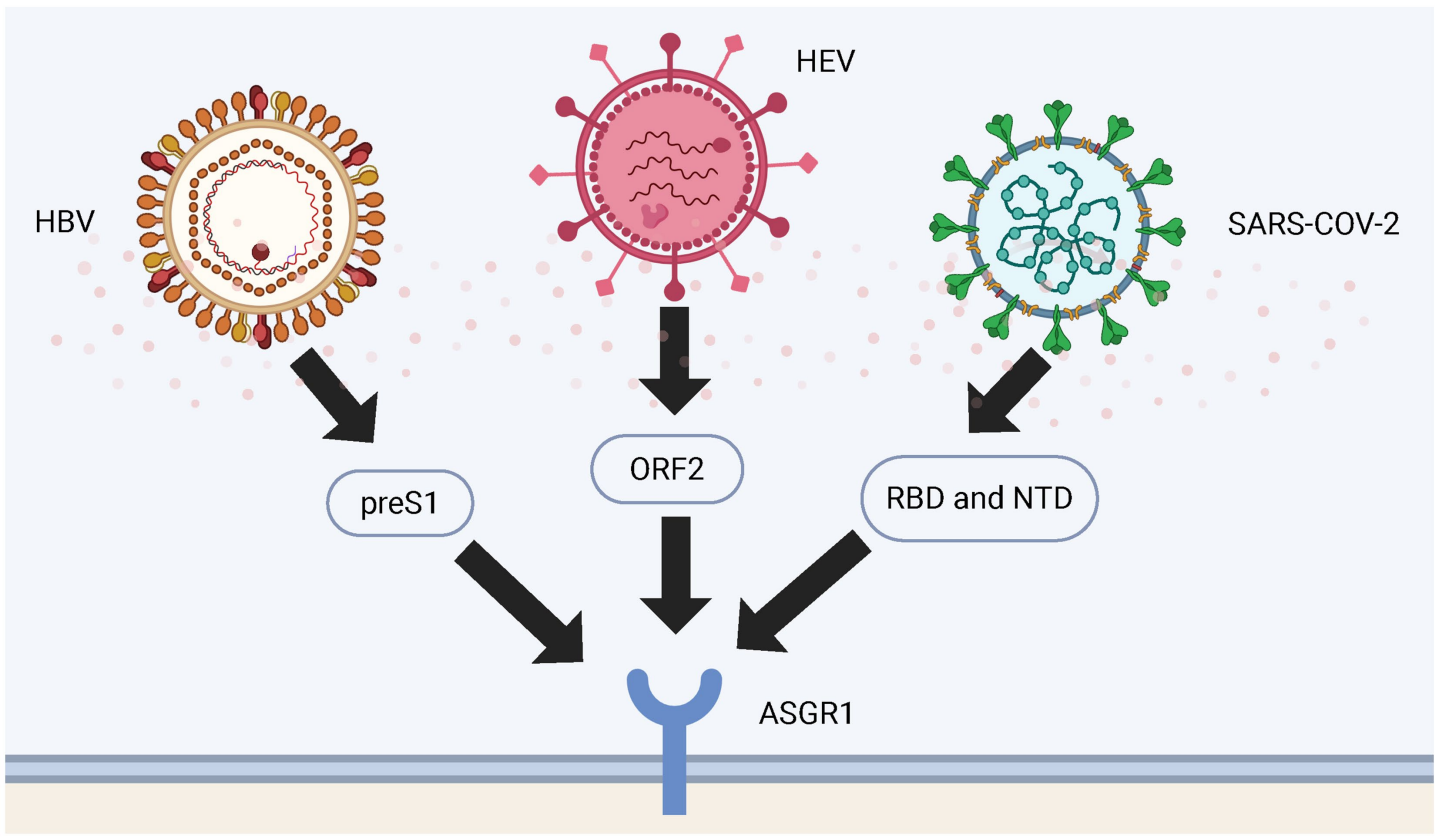
Binding sites of ASGR1 with viruses. The figure shows the binding sites of HBV, HEV and SARS-CoV-2 viral particles to ASGR1 during entry into the human body. HBV binds to ASGR1 through the preS1 region of its large surface protein; HEV interacts with ASGR1 via its capsid protein ORF2; and SARS-CoV-2 engages ASGR1 through either the RBD or the NTD of its spike protein.

After the emergence of severe acute respiratory syndrome coronavirus 2 (SARS-CoV-2), further research revealed that ASGR1 mediates viral entry into various tissues, including liver and lungs (79). Multiple independent groups confirmed ASGR1 as an alternative receptor to Angiotensin-Converting Enzyme 2 (ACE2), mediating SARS-CoV-2 entry through interactions with the receptor-binding domain (RBD) and N-terminal domain (NTD) of the spike glycoprotein, promoting ACE2-independent cellular entry and resistance to certain antibodies (5, 80, 81). Particularly in hepatocytes, high ASGR1 expression markedly enhances SARS-CoV-2 binding and infection, suggesting ASGR1-mediated pathways contribute significantly to viral infection and pathogenic mechanisms (82). ASGR1 also participates in virus-induced inflammation and immune modulation, potentially collaborating with other CLRs to exacerbate inflammation, enhancing viral-induced immunopathological damage (83) (Figure 3).

Studies have found that HBV, hepatitis C virus (HCV), and SARS-CoV-2 can partially rely on ASGR1-mediated glycan recognition to facilitate their attachment to and entry into hepatocytes (76, 78, 82). This mechanism may provide important insights into the molecular basis of viral hepatotropism, inform the development of glycan-targeted antiviral strategies, and guide the design of ASGR1-based liver-targeting viral vectors. Given that multiple viruses can exploit ASGR1 to mediate cellular entry, future research may explore the development of broad-spectrum ASGR1 inhibitors to block diverse viral infection pathways. Moreover, from the perspective of genetic polymorphism, identifying expression variations or splicing isoforms of ASGR1 through techniques such as single-cell RNA sequencing (84) and long-read transcriptome analysis (85) may help predict individual susceptibility to viral infections, offering new insights for precision prevention and control strategies.

### 3.3 The role of ASGR1 in tumors

Hepatocellular carcinoma (HCC) is one of the most common primary liver malignancies, characterized by high invasiveness and poor prognosis, representing a significant global health burden. The primary risk factors for HCC vary by region. In most high-risk areas, chronic HBV infection is the main contributor. In regions with lower HCC incidence, the rising number of cirrhosis patients largely explains the increasing HCC rates, driven by factors such as higher HCV-related cirrhosis incidence, HBV-related cirrhosis (to a lesser extent), and improved overall survival among cirrhosis patients (86).

Tumor cells typically exhibit highly sialylated glycan structures on their surfaces, a feature that facilitates immune evasion by preventing recognition and clearance by the immune system (87). In most tumor tissues, truncated O-glycans are abnormally overexpressed (88). The Tn antigen, a type of immature O-glycan, consists of a single N-acetylgalactosamine (GalNAc) residue attached to the serine (Ser) or threonine (Thr) residue of a protein, and is classified as one of the truncated O-glycans (88, 89). The Tn antigen promotes immune tolerance by engaging with immunosuppressive receptors, thereby contributing to tumor immune evasion (90). Notably, its structural characteristics closely resemble the ligand recognition pattern of ASGR1 (2, 3). Whether ASGR1 can effectively recognize the Tn antigen and mediate relevant biological functions remains to be further investigated. It is worth noting that the antigenicity of Tn has been validated in mice; antibodies induced by Tn-modified glycopeptides have been shown to effectively inhibit the growth of transplanted tumor cells, thereby exerting protective anti-tumor effects (91).

Research indicates that ASGR1 exerts tumor-suppressive effects by inhibiting phosphorylation of the STAT3 signaling pathway. Specifically, ASGR1 interacts with Nemo-like kinase (NLK) to inhibit glycoprotein 130/janus kinase 1 (GP130/JAK1)-mediated STAT3 phosphorylation, thereby blocking the activation of STAT3-related pro-tumor signaling pathways (92). Additionally, ASGR1 interacts with longevity assurance homolog 2 (LASS2) to suppress vacuolar-type H^+^-ATPase (V-ATPase) activity, reducing HCC cell migration and invasion, further supporting the role of ASGR1 as a metastasis suppressor in liver cancer (93). Multiple studies have reported significant downregulation of ASGR1 expression in HCC tissues, with expression levels decreasing further as tumor progression and grading advance (94). ASGR1 mRNA and protein levels are markedly reduced in Edmondson grade III-IV HCC tissues (95).

In addition, based on liquid biopsy technology, recent studies have analyzed the phenotypic and genetic markers of ASGR1 expression in circulating epithelial cells (CECs) from the peripheral blood of patients with liver cirrhosis (LC) and HCC. Findings reveal that ASGR1-negative CECs significantly increase the risk of developing HCC, particularly strong predictive power in patients with pre-existing LC. Further analysis revealed that ASGR1 expression in CECs remains detectable in the early stages of HCC, such as in LC patients with lower Child-Pugh scores, suggesting its potential as a non-invasive tool for early tumor monitoring. In addition, the combined loss of ASGR1 and miR-122-5p in CECs indicates a dedifferentiation tendency of tumor cells and is closely associated with poorer progression-free survival (PFS). Collectively, this study proposes that ASGR1 holds promise as a precise biomarker for early risk stratification and prognostic assessment in HCC, with potential application in cancer interception (96).

Meanwhile, ASGR1^+^ tumor-associated microparticles (taMPs) are significantly elevated in the peripheral blood of patients with HCC and cholangiocarcinoma, suggesting their potential as a novel non-invasive diagnostic tool for distinguishing liver cancer from liver cirrhosis (94). ASGR1 expression is also closely associated with immune cell infiltration, including B cells, CD8^+^ T cells, and DCs, indicating its role in tumor microenvironment immune modulation. A low methylation level in the promoter region is positively correlated with low ASGR1 expression, suggesting that its expression is influenced by epigenetic regulation (6).

**Figure 4:**
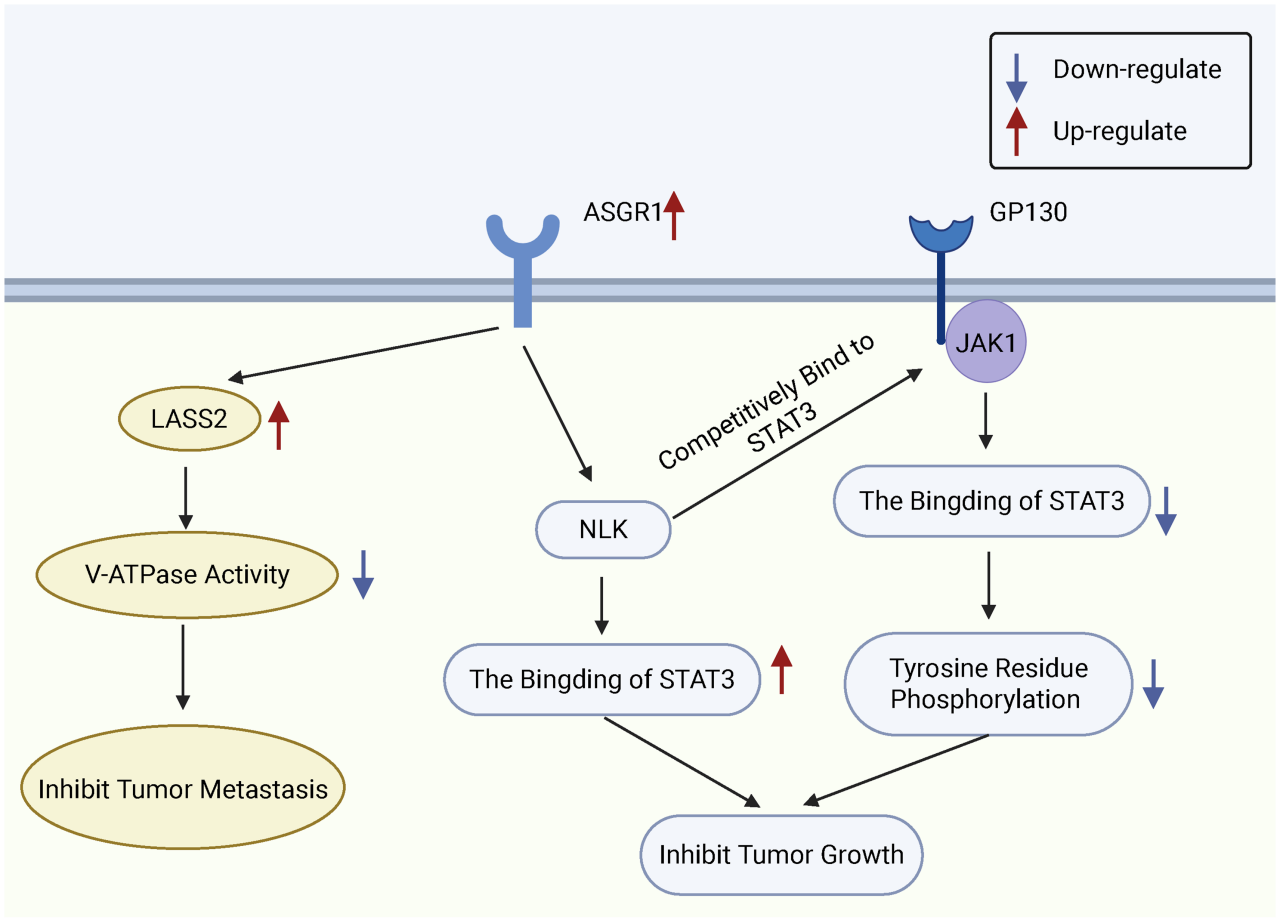
Functional role of ASGR1 in HCC cells. ASGR1 inhibits the metastasis and growth of hepatocellular carcinoma. ASGR1 mediates the inhibition of V-ATPase activity through LASS2, thereby suppressing HCC metastasis. Additionally, ASGR1 inhibits HCC progression by promoting the interaction between NLK and STAT3 and suppressing STAT3 phosphorylation.

Collectively, these findings underscore the potential of ASGR1 as a biomarker for HCC diagnosis and prognosis. Beyond acting as a tumor suppressor during HCC development, ASGR1 expression on circulating cells may serve as an effective liquid biopsy marker for early screening, risk stratification, and treatment monitoring. Future large-scale cohort studies are needed to validate its clinical translational value (Figure 4).

### 3.4 The role of ASGR1 in lipid metabolism

The clearance of low-density lipoprotein cholesterol (LDL-C) from plasma is primarily mediated by low-density lipoprotein receptors (LDLR) on hepatocyte surfaces (97). Proprotein convertase subtilisin/kexin type 9 (PCSK9), highly expressed in the liver, binds LDLR non-enzymatically after being secreted into the plasma, promoting LDLR degradation within cells, thereby reducing LDLR levels on hepatocyte surfaces and ultimately increasing plasma LDL-C levels (98–100). Interestingly, like PCSK9, ASGR1 can also directly target LDLR. Both proteins independently interact with LDLR and promote its degradation, thus regulating the number of LDLRs on hepatocyte surfaces and influencing plasma LDL-C levels (101). Studies have shown that in ASGR1-deficient conditions, PCSK9 expression at both mRNA and protein levels is significantly reduced (102). Further research indicates that the increased LDL uptake in ASGR1-deficient hepatocytes is associated with decreased PCSK9 levels, suggesting that the enhanced LDL uptake may result from stabilized LDLR due to reduced PCSK9 (102, 103). However, it is currently only known that ASGR1 may indirectly affect LDLR stability, while its precise regulatory mechanisms on PCSK9 expression, secretion, or function have not been fully elucidated and require further scientific investigation.

**Figure 5:**
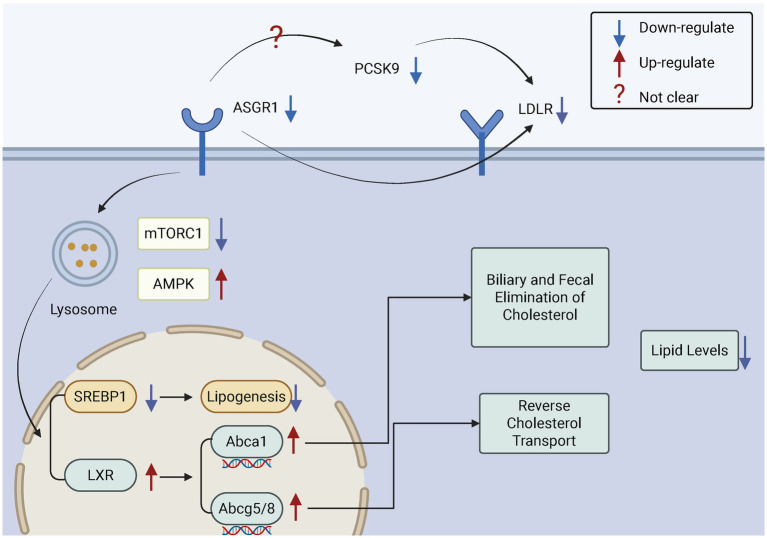
The role of ASGR1 in cholesterol regulation. ASGR1 regulates cholesterol excretion in bile and feces and modulates reverse cholesterol transport, thereby controlling lipid levels. Inhibition of ASGR1 upregulates LXRα, ABCA1, and ABCG5/G8, suppresses SREBP1, and reduces lipogenesis, which promotes cholesterol excretion and lowers lipid levels.

Studies have shown that ASGR1 deficiency increases the expression of insulin-induced gene 1 (INSIG1), thereby inhibiting sterol regulatory element-binding proteins (SREBPs) and reducing cholesterol synthesis (102). Similarly, a study by the team of Song Baoliang at Wuhan University reported comparable findings. Specifically, they demonstrated that ASGR1 deficiency suppresses mechanistic Target of Rapamycin Complex 1 (mTORC1) activity and activates 5’-AMP-activated protein kinase (AMPK), leading to reduced expression of the liver X receptor alpha (LXRα) ubiquitin ligase complex breast cancer type 1 susceptibility protein/BRCA1 associated ring domain protein 1 (BRCA1/BARD1). This reduction decreases the ubiquitination and degradation of LXRα, resulting in elevated LXRα levels. The upregulation of LXRα subsequently increases the expression of cholesterol transport-related genes such as ATP-binding cassette transporter A1 (ABCA1) and ATP-binding cassette sub-family G members 5 and 8 (ABCG5/G8), promoting cholesterol efflux and reducing plasma and hepatic lipid levels. In addition, ASGR1 deficiency suppresses SREBP1-mediated fatty acid synthesis, thereby reducing triglyceride (TG) production and hepatic lipid accumulation, contributing to the maintenance of liver metabolic homeostasis. Furthermore, ASGR1 is also involved in lipoprotein metabolism. It may play a role in high-density lipoprotein (HDL)-mediated cholesterol efflux, and its deficiency appears to enhance HDL-mediated cholesterol uptake and promote bile acid synthesis (48) (Figure 5).

Beyond regulating lipid metabolism, ASGR1 is also closely associated with lipid redistribution and transport. ASGR1 deficiency affects lipid distribution in adipose tissue. In obesity, ASGR1-deficient mice show increased visceral adipose tissue (VAT) lipid accumulation but decreased plasma lipid levels, potentially due to ASGR1-mediated lipid redistribution (104).

The role of ASGR1 in lipid regulation may have profound implications for atherosclerosis (AS) and cardiovascular disease (CVD) (7, 105). Independent studies conducted by multiple research groups have consistently demonstrated that ASGR1 knockout effectively reduces LDL levels and improves lipid profiles, indicating high reproducibility and mutual support of these findings (48, 49, 106). Moreover, studies in both pig and mouse models have consistently shown that ASGR1 deficiency improves lipid metabolism and attenuates atherosclerotic phenotypes, further confirming the cross-species consistency of these results (106, 107). Given its multiple roles in lipid regulation, ASGR1 may represent a novel therapeutic target for cardiovascular disease. Since ASGR1 deficiency can reduce non-HDL-C and lower CVD risk, small molecules or antibodies that inhibit ASGR1 may serve as promising new lipid-lowering agents (48).

However, some studies have shown that ASGR1 deficiency in ApoE^−^/^−^ mice leads to reductions in plasma cholesterol and triglyceride levels and alleviates atherosclerotic lesions. Nevertheless, this is also accompanied by an increase in immune cell populations and widespread alterations in hepatic metabolic pathways, despite the absence of overt liver morphological abnormalities (106). Similarly, ASGR1-deficient pigs fed a high-fat diet exhibit reduced non-HDL-C levels and attenuated atherosclerosis, with underlying mechanisms involving downregulation of cholesterol synthesis and enhanced clearance. However, mild to moderate liver injury was also observed (107).

Therefore, during the development of ASGR1-targeted therapeutics, close attention must be paid to the potential systemic metabolic remodeling and associated toxic effects, with comprehensive assessments of safety and tolerability (108). Although no apparent organ-specific lesions have been observed in current ASGR1-deficient animal models (106), the predominant hepatic expression of ASGR1 suggests that its loss of function may impair glycoprotein clearance and the stability of bile components (108, 109), potentially increasing the burden on the hepatobiliary system. Hence, as ASGR1 inhibitors—whether antibody-based or nucleic acid-based—progress toward clinical translation, their multisystem effects should be thoroughly evaluated to ensure effective lipid-lowering and anti-atherosclerotic activity while maintaining a favorable safety margin and therapeutic window.

## 4 ASGR1-based liver-targeted drug delivery

In the 1980s, studies on binding specificity laid the foundation for ASGP-R–targeted design (2, 3). Baenziger and Lee independently discovered that ASGP-R exhibits significantly higher affinity for GalNAc than for galactose (110, 111). In 1995, Lee and Ts’o first reported the successful conjugation of trivalent GalNAc to oligonucleotides, which effectively inhibited viral DNA expression—marking a key breakthrough in GalNAc-targeted delivery technology (112, 113).

GalNAc-siRNA and GalNAc-ASO are liver-targeted nucleic acid drug platforms developed based on N-acetylgalactosamine (GalNAc) conjugation technology. They achieve gene silencing through RNA interference (RNAi) and antisense mechanisms, respectively, and are used to treat various liver-related diseases (114, 115). Currently, a variety of GalNAc-conjugated therapeutics are under development (Table 1), with a substantial number having entered clinical trials or even reached market approval. These include nucleic acid-based drugs, liposomal formulations, and antibody-based therapies, among others (Table 2).

**Table 1:** Preclinical-stage therapeutic candidates based on GalNAc-conjugation technology.

Main technology types	Drug name	Target	Indication/application	Current stage	References
GalNAc-siRNA	Not specified	*Xor*	HUA	Preclinical (mouse models)	Sun et al. (134)
	Not specified	TKT	MAFLD	Preclinical (mouse models)	Tong et al. (135)
GalNAc-ASO	Not specified	*TMPRSS6* (Matriptase-2)	MASLD	Preclinical (MASLD mouse model)	Pettinato et al. (136)
	Not specified	SAB	JNK-dependent liver injury	Preclinical (mouse model)	Win et al. (137)
GalNAc-SSO	Not specified	*SLC25A13* c.469-2922G > T	CD	Preclinical (mouse model)	Ow et al. (138)
	Not specified	*PAH* mis-splicing variants	PKU	Preclinical (CRISPR/Cas9-edited cell model)	Martínez-Pizarro et al. (139)
GalNAc-liposome	Tn-Lipo-PTX	ASGR1	HCC	Preclinical (cellular validation)	Li et al. (140)
GalNAc-antibody	Alirocumab-tri-GalNAc	PCSK9	CVD	Preclinical (cell-based)	Donahue et al. (125)
	Cetuximab-tri-GalNAc	EGFR	Targeted degradation of membrane proteins	Preclinical (cell-based)	

*Xor*, Xanthine oxidoreductase; HUA, Hyperuricemia; TKT, Transketolase; MAFLD, Metabolic dysfunction-associated fatty liver disease; MASLD, Metabolic dysfunction-associated steatotic liver disease; SAB, SH3 domain-binding protein 5; CD, Citrin deficiency; PAH mis-splicing variants, phenylalanine hydroxylase gene mis-splicing variants; PKU, Phenylketonuria; PTX, PEGylated paclitaxel; HCC, Hepatocellular carcinoma; PCSK9, Proprotein Convertase Subtilisin/Kexin Type 9; CVD, Cardiovascular diseases; EGFR, Epidermal Growth Factor Receptor.

**Table 2:** Overview of GalNAc-conjugated nucleic acid drugs and their clinical indications.

Main technology types	Drug name	Originator company	Status	Target disease	References
GalNAc-siRNA	Inclisiran	Alnylam Pharmaceuticals Inc.	Launched	PH, FH, HC, ASCVD, HeFH	Lamb (141), Soffer et al. (142), Al Shaer et al. (143)
	Givosiran	Alnylam Pharmaceuticals Inc.	Launched	AHP	Scott (144)
	Lumasiran	Alnylam Pharmaceuticals Inc.	Launched	PH1	Scott and Keam (145)
	Vutrisiran	Alnylam Pharmaceuticals Inc.	Launched	FAP, SD	Keam (146)
	Nedosiran	Dicerna Pharmaceuticals Inc.	Launched	PH1	Syed (147)
	VIR-2218	Vir Biotechnology Inc.	Phase II clinical trial	CHB	Yuen et al. (148)
GalNAc-ASO	Bepirovirsen	GlaxoSmithKline/Ionis Pharmaceuticals Inc.	Phase II clinical trial	CHB	Yuen et al. (149)
	Olezarsen	Ionis Pharmaceuticals Inc.	Launched	FCS	Syed (150)
	Volanesorsen	Akcea Therapeutics, Inc.	Launched	FCS, FPL	Paik and Duggan (151)
GalNAc-SSO	Casimersen	Sarepta Therapeutics, Inc.	Launched	DMD	Shirley (152)
	Viltolarsen	Nippon Shinyaku, Inc. (in collaboration with NCNP)	Launched	DMD	Dhillon (153)

PH, Primary Hypercholesterolemia; FH, Familial Hypercholesterolemia; HC, Hypercholesterolemia; ASCVD, Atherosclerotic Cardiovascular Disease; HeFH, Heterozygous Familial Hypercholesterolemia; AHP, Acute Hepatic Porphyria; PH1, Primary Hyperoxaluria; FAP, Familial amyloid neuropathy; SD, Stargardt disease; CHB, Chronic Hepatitis B; FCS, Familial Chylomicronemia Syndrome; FPL, hypertriglyceridemia and familial partial lipodystrophy; DMD, Duchenne muscular dystrophy; NCNP, National Center of Neurology and Psychiatry.

Owing to the highly specific expression of ASGR1 on the surface of hepatocytes and its efficient endocytic capability, this receptor has been extensively utilized in the development of targeted therapeutic strategies for viral infections, particularly for the precision treatment of chronic hepatitis B (CHB) (116).

The GalNAc-siRNA platform, notably VIR-2218, effectively reduces hepatitis B virus surface antigen (HBsAg) expression, exhibiting strong antiviral potential (116). Several GalNAc-ASO drugs, such as Bepirovirsen and RO7062931, have advanced to clinical trials for chronic hepatitis B (CHB), demonstrating effective HBsAg clearance (117, 118). Building on this, recent studies have further explored the delivery of ASO drugs featuring a bivalent bile acid structure via the ASGR1 pathway. This strategy significantly enhances liver-targeted drug delivery and holds promise for improved clinical efficacy (119). GalNAc-LNA-SSO, leveraging the locked nucleic acid (LNA) and gapmer design, can effectively reduce HBV mRNA and HBsAg expression levels. It is recommended to combine this strategy with immune enhancers or polymerase inhibitors to achieve complete viral clearance (120). Moreover, the design of pan-genotypic targets for different HBV genotypes may represent a key advantage and a promising future direction for this approach.

Beyond viral infections, the therapeutic potential of ASGR1 in cancer treatment is also gradually emerging. For instance, small-molecule drugs conjugated with Gal or GalNAc, such as betulin, can specifically recognize and enter ASGR1-expressing liver cancer cells, exerting targeted anti-tumor effects through mechanisms like oxidative stress (121). Additionally, glycopolymer-based nanocarriers mediated by ASGR1 have been designed to precisely deliver suicide gene therapies in combination with chemotherapeutic drugs, effectively inhibiting liver cancer cell growth and showing promising clinical potential (122). For example, using Gal/GalNAc-mediated delivery, chemotherapeutic agents like doxorubicin or paclitaxel are encapsulated in nanoscale extracellular vesicles (exosomes) that are specifically taken up by hepatocytes through ASGR1-mediated endocytosis (123). This strategy enhances drug specificity and stability, reduces systemic toxicity and side effects associated with traditional chemotherapy, and increases drug accumulation in liver cancer cells, thereby improving anti-tumor efficacy. Moreover, a carrier-free self-assembling nanomedicine strategy using celastrol-galactose nanoparticles (CE-Gal-NPs) has been proposed. This approach utilizes the galactose recognition capability of ASGR1 to achieve liver cancer cell targeting and exerts potent anti-cancer effects by inducing ferroptosis while significantly reducing toxicity (124). Considering the variability of ASGR1 expression among liver disease patients, it is recommended to use companion diagnostics to stratify patients during clinical application of ASGR1-targeted therapies, thereby improving therapeutic outcomes (95).

Furthermore, with the advancement of targeted protein degradation technologies, ASGR1, as a key endocytic receptor, enables the selective degradation of extracellular or membrane proteins, thereby expanding its application potential in precision medicine (125).

Targeted protein degradation (TPD) has become an important strategy for studying protein degradation mechanisms and developing new treatments for diseases (126). Most current TPD platforms, such as immunomodulatory imide drugs (IMiDs) (127, 128) and proteolysis targeting chimeras (PROTACs) (129, 130), rely on the intracellular ubiquitin–proteasome system (UPS), making it difficult to target membrane-bound or extracellular proteins. To address this limitation, RME has been introduced into TPD design.

ASGR1, a highly expressed endocytic receptor in hepatocytes, recognizes specific glycans and efficiently mediates endocytosis, making it a critical component of lysosome-targeting chimeras (LYTACs). LYTACs link trivalent GalNAc ligands to target proteins, enabling ASGR1 to recognize and deliver them to lysosomes for degradation, thereby allowing selective regulation of extracellular or membrane proteins (131).

In addition, the emerging EndoTags (endocytosis-triggering binding proteins) technology utilizes engineered protein modules to induce endocytosis without relying on natural ligands, offering greater flexibility in target selection and broader applicability (132). However, ASGR1, being naturally expressed and highly liver-specific, may offer advantages in minimizing immunogenicity and off-target effects.

Looking ahead, integrating the functional properties of ASGR1 with tools from synthetic biology to achieve multilevel regulation may enable the precise identification and degradation of liver disease–related proteins, thus expanding the therapeutic potential of ASGR1-based strategies in liver disorders.

## 5 Extrahepatic functions of ASGR1

Multiple studies have identified ASGR1 homologs or functionally similar receptors in various non-hepatic cell types, suggesting that the biological roles of ASGR1 may be far more extensive than previously understood (50, 51).

Studies have identified a human dendritic cell isoform of ASGR1 (DC-ASGPR) with a lectin domain structurally similar to hepatic ASGR1. Its expression decreases during DC maturation, suggesting a role in antigen uptake and immune regulation. Functional assays confirm its endocytic capability, extending the expression of ASGR1 beyond hepatocytes to include DCs involved in immune uptake (52).

Caco-2 cells endogenously express a functional hepatic-type ASGR1 receptor, with both H1 and H2 subunits specifically recognized by subunit-specific antibodies. The receptor is primarily localized to the basolateral membrane, similar to its distribution in hepatocytes. Compared to HepG2 cells, ASGR1 in Caco-2 cells exhibits slight differences in glycosylation patterns, although the mRNA size remains identical, indicating no significant transcript variation. The H1 subunit is the predominant component in both cell types. These findings suggest that Caco-2 cells not only resemble hepatocytes in structure but also functionally mimic hepatic ASGR1 behavior (54). Human RPTEC endogenously express functional ASGR1, with both H1 and H2 subunits detected at the mRNA and protein levels, primarily localized to the cytoplasm and basolateral membrane. In contrast, the human renal epithelial cell line human kidney-2 (HK2) expresses only ASGR1 H1 subunit mRNA, suggesting limited functional capacity. Functional assays demonstrated that RPTEC specifically bind and internalize the ASGR1 ligand, asialofetuin, confirming the receptor’s characteristic ligand recognition and endocytic activity, and providing strong evidence for the presence and functionality of ASGR1 in the kidney (55).

The expression of ASGR1 in human testis is significantly lower than in the liver, with primary localization in the seminiferous tubules and interstitial regions. It is specifically expressed in Sertoli cells and Leydig cells, while expression in spermatogonia is minimal. Multiplex immunohistochemical staining revealed co-localization of ASGR1 with vimentin, confirming its presence in Sertoli cells, while no co-localization was observed with the spermatogonial marker proliferating cell nuclear antigen (PCNA), suggesting weak expression of ASGR1 in germ cells (133). However, no further investigation has been conducted on the functional role of ASGR1 in the testis, such as whether it mediates endocytosis or glycoprotein clearance. Whether ASGR1 is directly involved in the HBV entry process remains speculative and lacks functional validation. Rat testes express a cell surface galactosyl receptor (RTG-r) that is antigenically similar to the minor hepatic ASGR isoforms RHL-2/3, but shows no cross-reactivity with the major isoform RHL-1. Affinity chromatography and SDS-PAGE analyses revealed that RTG-r shares the same electrophoretic mobility as RHL-2/3. RTG-r is consistently expressed throughout testicular development, localized in the seminiferous tubules, cultured Sertoli cells, and spermatogenic cells, and is also present on the surface of epididymal sperm, particularly concentrated on the dorsal region of the sperm head plasma membrane. Given its galactose-binding ability and distribution in meiotic and post-meiotic spermatogenic cells as well as the acrosomal region of sperm, RTG-r is suggested to play a role in spermatogenesis and sperm–egg recognition (56).

The major subunit of the rat hepatic ASGPR, RHL-1, is not only highly expressed on the basolateral membrane of hepatocytes, but is also present in thyroid tissue and differentiated thyroid cell lines. Its expression can be upregulated by thyroid-stimulating hormone (TSH), whereas neoplastic transformation leads to its downregulation. Immunohistochemical analysis shows that RHL-1 is localized to the apical membrane of thyroid cells, contrasting with its basolateral localization in hepatocytes, suggesting tissue-specific membrane distribution. Although its expression level in the thyroid is relatively low, RHL-1 is still capable of binding asialoorosomucoid (ASOR) and thyroglobulin (Tg), and mediating their endocytosis, indicating a potential role in Tg lysosomal trafficking and regulation of thyroid hormone processing (53). However, no further explanation has been provided as to why RHL-1 in the thyroid, compared to the liver, appears to be more specialized in handling Tg, and whether structural or post-translational modifications contribute to this functional difference remains unknown.

These findings on extrahepatic ASGR1 largely lack systematic comparisons of structural, functional, or regulatory mechanisms between hepatic and extrahepatic ASGR1, and comprehensive functional validation is still needed. Therefore, the physiological significance of extrahepatic ASGR1 remains controversial and uncertain.

## 6 Conclusion

From the initial receptor-ligand recognition to the activation of downstream ASGR1 signaling, from receptor-mediated pathogen endocytosis to lipid metabolism regulation, and from hepatic to extrahepatic functions, the understanding of the biological functions of ASGR1 has evolved over a long journey. At present, the roles and mechanisms of ASGR1 in the development of viral infectious diseases, tumors, atherosclerosis, and other diseases have been well recognized. Clinical intervention strategies based on ASGR1, such as liver-targeted drug delivery and lipid-lowering treatments, are gradually showing trends toward clinical translation. Referring to the past exploration of the biological functions of lectin receptors, further investigation of the intrahepatic and extrahepatic functions of ASGR1 is the future direction of this research field.
